# Atrial Fibrillation and Cognitive Decline: A Systematic Review of Pathophysiological Mechanisms, Therapeutic Strategies, and Digital Health Technologies in Neuroprotection

**DOI:** 10.3390/jcm15051744

**Published:** 2026-02-25

**Authors:** Amparo Santamaria, Cristina Antón, Nataly Ibarra, María Fernández, Pedro González, Rafael Carrasco

**Affiliations:** 1 Hematology Department and Foundation for the Promotion of Health and Biomedical Research (FISABIO), University Vinalopó Hospital, 03293 Elche, Spain; camaldonado@vinaloposalud.com (C.A.); niibarra@vinaloposalud.com (N.I.); fndz.lopez.maria@gmail.com (M.F.); 2 Managemnet Department, Vinalopó University Hospital, 03023 Alicante, Spain; pgcabezas@vinaloposalud.com (P.G.); rcarrasco@vinaloposalud.com (R.C.)

**Keywords:** atrial fibrillation, cognitive impairment, dementia, silent cerebral infarction, cerebral hypoperfusion, cerebral microembolism, direct oral anticoagulants, catheter ablation, digital health, mHealth platforms, neuroprotection, heart–brain health

## Abstract

**Background**: Atrial fibrillation (AF) is consistently associated with cognitive impairment and dementia through mechanisms that extend beyond classical cardioembolic stroke. However, the relative contribution of these pathways and the effectiveness of available therapeutic strategies for preserving cognition remain uncertain, as most data come from observational studies with a substantial risk of bias. **Objectives**: This review narratively synthesizes contemporary evidence on epidemiology, pathophysiological mechanisms, therapeutic strategies—including anticoagulation, rhythm control, and comprehensive risk-factor management—and the role of digital health technologies in the relationship between AF and cognitive decline. **Methods**: We performed a narrative, PRISMA-informed scoping review of observational cohorts, mechanistic studies, randomized clinical trials, systematic reviews, and meta-analyses published up to January 2026, identified through structured searches in MEDLINE/PubMed and complementary sources. Studies were selected if they examined (i) associations between AF and cognitive impairment or dementia, (ii) mechanistic pathways linking AF to brain injury, (iii) therapeutic interventions with cognitive or brain imaging outcomes, or (iv) digital health technologies applied to AF management. Heterogeneity in study design and outcome assessment precluded meta-analysis; therefore, we provide a qualitative synthesis, explicitly distinguishing observational evidence from randomized data and discussing key sources of confounding. Risk of bias was evaluated using validated tools: ROBINS-I for non-randomized studies, RoB 2.0 for RCTs, Newcastle–Ottawa Scale for observational cohorts, and AMSTAR-2 for systematic reviews. **Results**: Large population-based cohorts and meta-analyses indicate that AF is associated with a 1.4–2.2-fold higher risk of cognitive impairment or incident dementia, even after adjustment for shared vascular risk factors and exclusion of patients with prior stroke; nevertheless, residual confounding and selection bias cannot be excluded. Silent cerebral infarcts are detected in roughly one-quarter to two-fifths of AF patients without clinical stroke and are themselves associated with cognitive deficits, suggesting that subclinical embolism represents one important, but not exclusive, pathway. Additional mechanisms include chronic cerebral hypoperfusion, neuroinflammation, small vessel disease, and structural brain atrophy, all of which are incompletely disentangled from comorbidities. Observational data suggest that oral anticoagulation, particularly with direct oral anticoagulants (DOACs), is associated with lower rates of dementia compared with no anticoagulation or warfarin, but randomized trials such as BRAIN-AF and GIRAF have not demonstrated a clear cognitive benefit, underlining the low-to-moderate certainty of this evidence. Rhythm-control interventions, especially catheter ablation, are associated with lower dementia incidence in registry studies, yet strong selection effects and short follow-up limit causal inference. Digital health tools and ABC-pathway mobile applications improve cardiovascular outcomes and adherence, although cognitive endpoints remain largely unexplored. **Conclusions**: AF should be conceptualized as a neurovascular condition with important implications for brain health, rather than a purely cardiac rhythm disorder confined to stroke prevention. A comprehensive heart–brain management strategy that combines optimal anticoagulation, individualized rhythm control, aggressive vascular risk factor modification, routine cognitive screening in older or high-risk patients, and judicious use of digital health technologies may offer the best opportunity for preserving cognition, although rigorous trials with cognitive endpoints are still needed to establish causality.

## 1. Introduction

### 1.1. Epidemiological Burden and Clinical Significance

Atrial fibrillation and dementia represent twin epidemics in an aging population. AF affects approximately 50–100 million individuals worldwide, with prevalence projected to increase substantially in the coming decades due to demographic shifts toward older populations. Similarly, dementia affects over 55 million people globally, with incidence doubling every 5 years after age 65. The intersection of these conditions presents a formidable public health challenge, yet the AF–dementia relationship remains understudied relative to well-established AF–stroke associations [1,2,3,4,5,6,7,8,9,10,11,12,13,14,15,16].

Traditionally, AF-related cognitive impairment has been attributed predominantly to cardioembolic stroke, which accounts for 20–30% of ischemic strokes in AF patients [1,2,3,4,5]. However, compelling epidemiological evidence over the past decade has demonstrated that AF is independently associated with cognitive decline and dementia, even in the absence of clinically recognized stroke. Large population-based cohort studies and meta-analyses consistently report that AF increases the relative risk of cognitive impairment and incident dementia by 1.4–2.2 fold, with the association persisting after adjustment for shared cardiovascular risk factors (hypertension, diabetes, heart failure, smoking, advanced age) and exclusion of patients with prior clinical stroke. The population-attributable risk for dementia resulting from AF is estimated at approximately 13%, suggesting that effective AF management and prevention strategies could meaningfully reduce the population burden of dementia [1,2,3,4,5,6,7,8,9,10,11,12,13,14,15,16].

### 1.2. Limitations of the Traditional Paradigm

The current clinical paradigm for AF management has focused almost exclusively on: (1) stroke risk stratification using tools such as the CHA_22_-VASc score, (2) anticoagulation initiation and optimization to reduce cardioembolic stroke, and (3) rhythm or rate control strategies to improve symptoms and prevent tachycardia-mediated cardiomyopathy. This stroke-centric approach, while appropriate and evidence-based, overlooks substantial subclinical brain injury and progressive cognitive deterioration occurring in many AF patients—particularly those considered “low-risk” by traditional stroke risk scores [6,7,8,9,10,11,12,13,14,15].

Recent neuroimaging and mechanistic studies have revealed that clinically recognized stroke represents “only the tip of the iceberg” of AF-induced brain ischemia. Silent cerebral infarcts (covert brain infarcts not accompanied by acute neurological symptoms) are detected on brain MRI in 25–40% of AF patients without prior clinical stroke—a prevalence substantially higher than the 1–5% annual clinical stroke rate. These silent infarcts are associated with measurable cognitive dysfunction and an increased future dementia risk. Beyond overt infarction, AF is associated with chronic cerebral hypoperfusion, neuroinflammation, accelerated cerebral small vessel disease, and structural brain atrophy—all potentially reversible through targeted intervention if identified early [6,7,8].

Together, these observations suggest that AF is better understood as a chronic neurovascular stressor than as an isolated cardiac arrhythmia whose cerebral consequences are limited to overt stroke. This broader perspective motivates a shift from a narrow, stroke-centric model toward an integrated heart–brain framework that considers cognitive trajectories, subclinical brain injury, and modifiable systemic factors as central treatment targets [10,11,12,13,14,15,16].

### 1.3. Rationale for a Comprehensive Review Approach

The complexity of multiple interacting pathophysiological mechanisms, combined with the heterogeneity of emerging therapeutic interventions and limited primary data specifically addressing cognitive endpoints, necessitates a comprehensive narrative review synthesizing evidence across epidemiology, mechanistic pathways, therapeutic domains, and digital health technologies. This integrative approach permits the identification of complementary therapeutic strategies operating through distinct mechanisms, recognition of critical knowledge gaps, and delineation of urgent research priorities to advance the field.

### 1.4. Central Thesis of This Review

The central thesis of this review is that AF should be considered a neurovascular disease that threatens brain health through several converging mechanisms and that clinical management must therefore expand beyond stroke prevention to include routine cognitive assessment and, in selected patients, rhythm-control strategies that may confer hemodynamic and potentially neuroprotective advantages. Rather than asserting that specific interventions prevent dementia, we critically appraise the extent to which current evidence supports associations between different therapeutic approaches and cognitive outcomes, and we highlight the many areas where causality remains unproven.

## 2. Materials and Methods

We conducted a PRISMA-informed narrative scoping review designed to synthesize the evidence linking atrial fibrillation (AF) with cognitive impairment, dementia, brain structural injury, and the impact of therapeutic and digital health interventions on neurocognitive outcomes. The review adhered to PRISMA 2020 guidance for transparent reporting of search procedures and study selection while recognizing that heterogeneity in study design and outcome measurement precluded quantitative pooling. Please refer to the Supplementary Materials, where the PRISMA checklist is available. 

### 2.1. Search Strategy and Data Sources

A structured literature search was performed in MEDLINE/PubMed, Embase, Scopus, and the Cochrane Central Register of Controlled Trials, covering the period January 2000 to January 2026. Additional records were identified through reference list screening, grey literature (conference abstracts, preprints), and expert recommendations. Search terms integrated both Medical Subject Headings (eSH) and free-text keywords, combining concepts related to AF, cognitive impairment, dementia, neuroimaging, mechanisms of brain injury, therapeutic interventions, and digital health technologies.

### 2.2. Eligibility Criteria

Studies were eligible if they met the following criteria:

1. Population: Adults (≥18 years) with documented AF.
2. Exposure/Intervention: AF diagnosis, anticoagulation, rhythm or rate control strategies, catheter ablation, or digital health approaches.
3. Comparators: Non-AF controls, alternative treatment strategies, or pre–post assessments.
4. Outcomes:
  - Validated cognitive measures (e.g., MoCA, MMSE, neuropsychological batteries);
  - Incident dementia (all-cause, Alzheimer’s, vascular);
  - Brain imaging markers (white matter hyperintensities, silent infarcts, brain atrophy).
5. Study Designs: Randomized controlled trials (RCTs), prospective or retrospective cohort studies, case–control studies, mechanistic studies, and systematic reviews with or without meta-analysis.

The following were excluded: animal or preclinical studies; paediatric populations; studies limited to stroke cohorts without broader AF assessment; publications lacking validated cognitive outcomes; non-English articles; conference abstracts without full data; editorials/commentaries; and low-quality studies (high risk of bias via ROBINS-I or Cochrane RoB 2.0).

6. Study Selection

After the removal of duplicates, titles and abstracts were screened independently by two reviewers. Full texts of potentially relevant articles were then evaluated. Conflicts were resolved by consensus. Studies were categorized into (i) observational cohorts, (ii) RCTs, (iii) mechanistic/pathophysiological studies, (iv) digital health interventions, and (v) systematic reviews/meta-analyses.

### 2.3. Data Extraction and Synthesis

Data were extracted using a standardized template including study design, sample characteristics, AF subtype, cognitive or imaging outcomes, follow-up duration, and main results. Because of substantial methodological heterogeneity—differences in cognitive assessments, AF ascertainment, adjustment for confounders, inclusion of stroke populations, and follow-up length—we performed a qualitative synthesis, explicitly distinguishing mechanistic from clinical outcome evidence.

### 2.4. Quality Assessment

**Figure 1:**
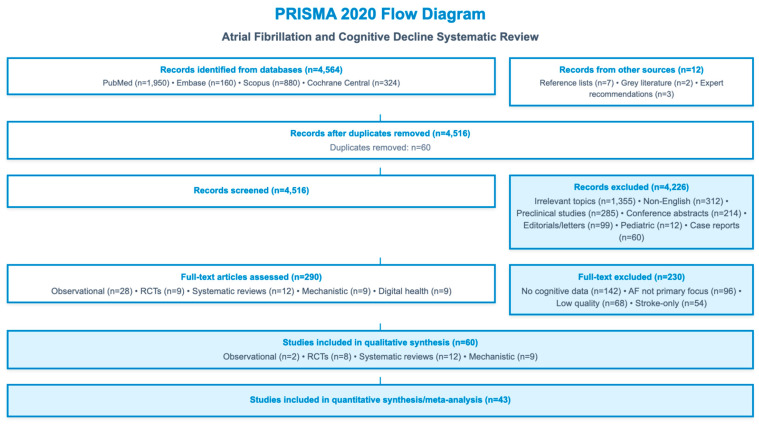
PRISMA 2020 flowchart diagram.

Risk of bias was evaluated using validated tools: ROBINS-I for non-randomized studies, RoB 2.0 for RCTs, Newcastle–Ottawa Scale for observational cohorts, and AMSTAR-2 for systematic reviews. Certainty of evidence was graded using the GRADE framework. See Figure 1.

## 3. Epidemiology of Atrial Fibrillation and Cognitive Impairment

### 3.1. Prevalence and Incidence of AF-Associated Cognitive Decline

Large population-based cohort studies have established that AF is independently associated with cognitive impairment across diverse populations. In the Framingham Heart Study, individuals with AF demonstrated significantly lower cognitive test scores and faster rates of cognitive decline compared to those without AF, with differences persisting after adjustment for age, education, cardiovascular risk factors, and prior stroke. Meta-analyses of observational studies consistently report that AF is associated with a 1.4–2.2 fold increased relative risk of incident dementia compared to individuals without AF, depending on the population studied, follow-up duration, and adjustment for confounders [9].

Although the consistency of these observational associations across cohorts and settings is striking, important limitations must be acknowledged. Most studies rely on administrative diagnoses or brief cognitive screening tools, are vulnerable to residual confounding by frailty, socioeconomic status, or access to care, and often lack detailed information on AF burden or neuroimaging markers, which limits the ability to draw causal conclusions.

### 3.2. Independent Association with Dementia Risk

Epidemiological studies have consistently demonstrated that the AF–dementia association is independent of shared cardiovascular risk factors. Adjustment for hypertension, diabetes, heart failure, smoking, obesity, and advanced age attenuates but does not eliminate the association between AF and dementia, providing evidence for AF-specific mechanisms beyond shared aetiology. This independence has been demonstrated across multiple cohorts using different dementia ascertainment methods (physician diagnosis, neuropsychological testing, neuroimaging criteria), supporting the specificity of the association [4].

Taken together, the available data support an independent association between AF and cognitive decline after adjustment for traditional vascular risk factors, but they do not prove that AF per se causes dementia. The observed relationships could partly reflect unmeasured comorbidities, reverse causation in very old populations, or treatment-related differences, underlining the need for carefully designed prospective studies with repeated cognitive assessments and comprehensive confounder control.

## 4. Pathophysiological Mechanisms Linking Atrial Fibrillation to Cognitive Decline

**Figure 2:**
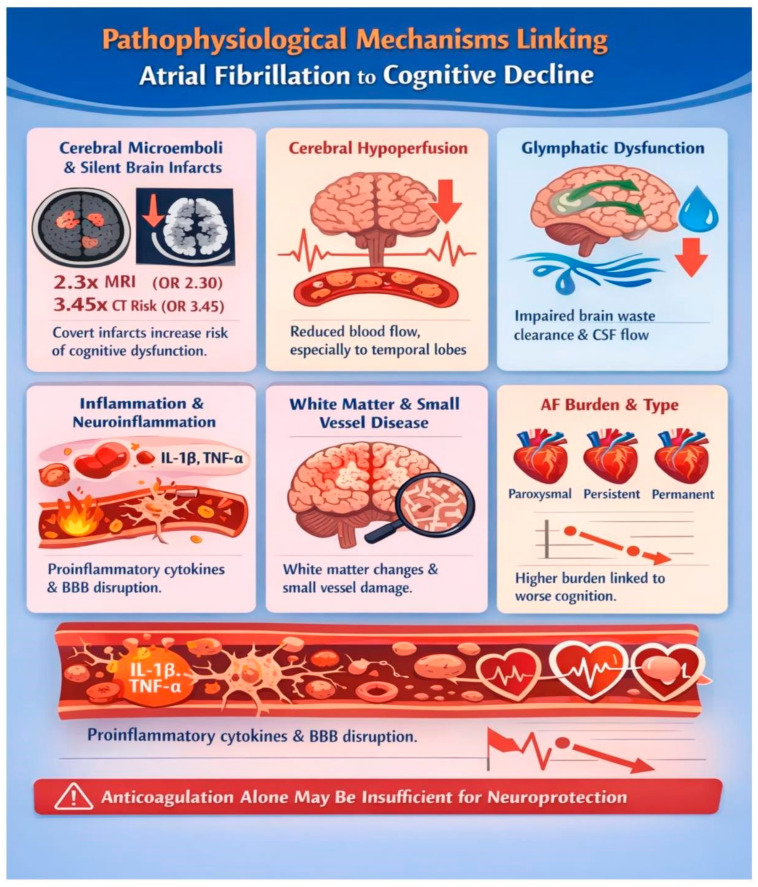
Pathophysiological mechanisms between atrial fibrillation and cognitive decline.

Several mechanisms are associated with the cognitive decline linked to atrial fibrillation (AF). Here, we outline some of these mechanisms. See Figure 2.

### 4.1. Cerebral Microembolism and Silent Brain Infarcts

Subclinical embolic brain ischemia (covert brain infarcts, cerebral microinfarcts) is likely the predominant mechanism accounting for cognitive deterioration associated with AF. A meta-analysis of 11 studies including 5317 AF patients and control subjects demonstrated that AF is associated with 2.30-fold increased odds of silent cerebral infarction on MRI (95% CI 1.44–3.68) and 3.45-fold increased odds of silent cerebral infarction on CT (95% CI 2.03–5.87).

The prevalence of silent cerebral infarction in AF patients is substantial, depending on the imaging diagnosis performed, for instance, 40% on MRI or 22% on CT, showing that the higher MRI detection rate reflects superior sensitivity for small infarcts compared to CT [6,7].

### 4.2. Chronic Cerebral Hypoperfusion

AF is associated with reduced cardiac output and cerebral blood flow. The irregular rate and loss of atrial contribution to ventricular filling compromise cardiac output, which may reduce cerebral perfusion, particularly in elderly patients with limited cerebrovascular autoregulation or concurrent cerebrovascular disease.

A landmark study using phase-contrast magnetic resonance imaging to measure blood flow velocity in the cervical arteries found striking differences between AF phenotypes (see Table 1).

These findings demonstrate that persistent AF results in a 15–20% reduction in total cerebral blood flow compared to sinus rhythm, with the greatest deficit in continuous arrhythmia [13,14,15].

### 4.3. Neuroinflammation and Systemic Inflammation

AF and Alzheimer’s disease share strikingly similar inflammatory profiles, both characterized by elevated systemic markers of inflammation (see Table 2).

The convergence of inflammatory profiles in AF and neurodegenerative disease suggests shared pathophysiological pathways linking cardiac arrhythmia to brain injury [13,14,15,16].

### 4.4. Brain Structural Changes and Atrophy

Multimodal neuroimaging studies have revealed that AF is associated with widespread structural brain alterations extending beyond focal vascular lesions. Analysis of 1335 stroke-free individuals with AF and 2683 matched controls using multimodal neuroimaging (structural MRI, diffusion tensor imaging, and free water imaging) revealed that AF patients exhibited a reduced cortical thickness in multiple brain regions, decreased grey matter volume in cortical and subcortical structures and also elevated extracellular free-water content, indicating neuroinflammation and neurodegeneration and widespread white matter abnormalities consistent with small vessel pathology [8]

Across these mechanistic domains—microembolism, hypoperfusion, inflammation, and structural brain changes—evidence remains largely circumstantial and predominantly observational. While it is biologically plausible that these processes mediate part of the AF–dementia relationship, the relative contribution of each pathway and their interaction with age-related neurodegeneration are still uncertain, and few studies have linked specific mechanistic markers to long-term cognitive trajectories in a causal framework [6,7,8,9,10,11,12,13,14,15,16].

## 5. Therapeutic Strategies for Cognitive Protection in Atrial Fibrillation

### 5.1. Anticoagulation and Cognitive Outcomes

Across multiple observational cohorts, oral anticoagulation (OAC) in atrial fibrillation (AF) is associated with lower rates of incident dementia compared with no anticoagulation, with relative risk reductions commonly reported in the ~40–60% range. Within OAC, direct oral anticoagulants (DOACs)—notably apixaban and rivaroxaban—are often associated with more favourable cognitive outcomes than warfarin, with pooled estimates suggesting an ≈30% lower dementia incidence among DOAC-treated patients (RR ≈ 0.69). These associations are biologically plausible (e.g., prevention of thromboembolism and microinfarction), but they warrant cautious interpretation because treatment allocation is non-random and DOAC users typically differ from VKA users in age, comorbidities, renal function, adherence, and access to care [9,17,18,19,20,21,22,23].

Consistent with these caveats, systematic reviews/umbrella analyses and GRADE assessments generally rate the certainty of evidence for a protective association between OAC—particularly DOACs—and reduced dementia risk as low to moderate [19,20,21]. Moreover, signals from randomized data are neutral. Trials such as BRAIN-AF [21] and GIRAF [19] have not demonstrated a significant cognitive advantage of DOACs over VKAs, arguing against a large direct neuroprotective effect in lower-risk, cognitively intact populations [1,2,10]. These trials were limited by relatively short follow-up, modest statistical power for small cognitive differences, and inclusion criteria that may under-represent patients at greatest risk of cognitive decline.

Taken together, OAC use is observationally associated with lower dementia risk, and DOACs may show a modest advantage over VKAs; however, causality remains unproven. The totality of evidence supports an associative interpretation, with moderate-to-low certainty, reflecting potential residual confounding, immortal-time bias, healthy-user effects, and heterogeneity in populations and outcome definitions. Anticoagulation remains fundamental for stroke prevention in AF, but its direct role in preventing cognitive decline has yet to be established. Larger trials focusing on high-risk or cognitively vulnerable patients, longer follow-up, and consistent neurocognitive measures are needed to determine if there is any added cognitive benefit beyond stroke reduction [9,10,17,18,19,20,21,22,23].

### 5.2. Rhythm Control and Hemodynamic Optimization

Restoration and maintenance of sinus rhythm offer several potential cognitive benefits through distinct physiological mechanisms that extend beyond simple symptom relief. By reinstating the atrial contribution to ventricular filling and eliminating the irregular ventricular response characteristic of atrial fibrillation (AF), rhythm control strategies can improve cardiac output and cerebral blood flow, addressing the chronic hypoperfusion documented in persistent AF. Additionally, suppression of AF episodes through successful rhythm control may reduce the formation of atrial thrombi and subsequent microemboli while simultaneously mitigating the systemic and brain inflammatory markers that are elevated during atrial remodelling and perpetuated by ongoing arrhythmia.

Observational evidence supporting these theoretical advantages comes primarily from population-based cohort studies examining catheter ablation outcomes. Data from large Korean and European registries suggest that AF patients who undergo successful catheter ablation experience 52–69% lower rates of incident dementia compared to those managed medically (HR 0.52–0.69). These findings align with the hemodynamic rationale outlined above and raise the possibility that rhythm control could confer cognitive benefits independent of stroke prevention.

Overall, the available evidence suggests that restoration and maintenance of sinus rhythm may be associated with better cognitive trajectories, particularly when AF burden and hemodynamic instability are substantial. However, most ablation studies are observational, include relatively young and carefully selected patients, and are at high risk of selection bias, making it premature to regard rhythm control as definitively neuroprotective. Future trials should incorporate standardized cognitive endpoints, neuroimaging, and detailed assessment of AF burden to clarify whether rhythm control modifies dementia risk beyond symptom relief [5,6].

### 5.3. Comprehensive Management of Cardiovascular Risk Factors 

Beyond anticoagulation and arrhythmia management, comprehensive control of modifiable cardiovascular risk factors represents a foundational element of cognitive protection in AF patients. Aggressive blood pressure management targeting <130/80 mmHg reduces both stroke risk and cognitive decline trajectories, while LDL cholesterol control below 70 mg/dL in high-risk individuals appears to exert direct neuroprotective effects. Similarly, individualized glycaemic control (HbA1c 7–8%) has been associated with cognitive benefits, complete smoking cessation prevents ongoing vascular injury, regular physical activity (≥150 min/week of moderate aerobic exercise) promotes cerebral blood flow, and systematic screening and treatment of sleep apnoea with CPAP addresses a condition bidirectionally linked to both AF and cognitive impairment.

This multi-domain approach recognizes that AF rarely occurs in isolation and that the cumulative burden of vascular risk factors likely amplifies the brain injury associated with arrhythmia through shared pathways of endothelial dysfunction, microvascular disease, and systemic inflammation [15,16].

## 6. Evolution of Digital Health in Managing Atrial Fibrillation

The story of digital health in atrial fibrillation (AF) care begins with a simple but powerful premise: the convergence of technology and medicine holds the potential to reshape cardiovascular treatment. As AF becomes increasingly common, the stakes of early detection and effective management—especially the prevention of stroke and cognitive decline—rise ever higher [23,24,25,26,27,28,29,30,31,32,33,34,35,36,37,38,39,40,41,42,43,44,45].

**Figure 3:**
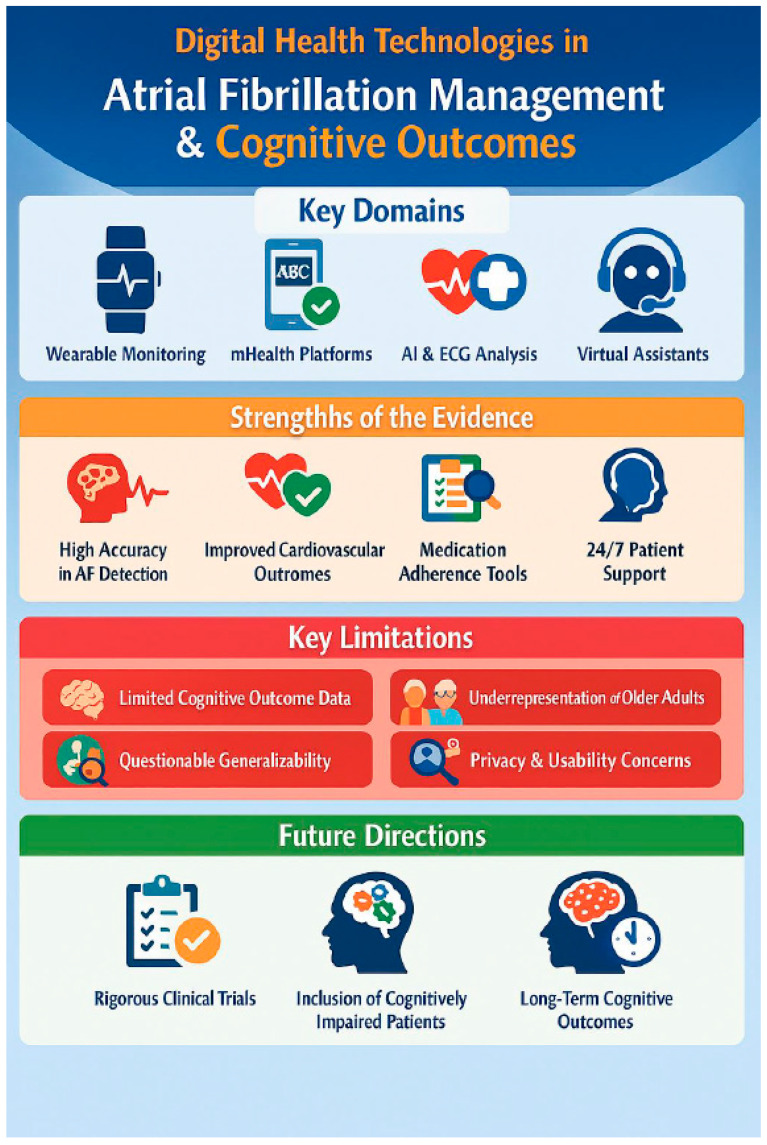
Digital Health Technologies in Atrial Fibrillation Management and Cognitive Outcomes.

The American Heart Association highlights that AF-related cognitive impairment arises from multifactorial pathways—stroke, microinfarction, cerebral hypoperfusion, and shared vascular risk factors—underscoring the importance of early identification and integrated management across the disease continuum [23,24,25,26,27,28,29,30,31,32,33,34,35,36,37,38,39,40,41,42,43,44,45]. Figure 3 provides an overview of the digital tools involved.

### 6.1. Wearables and Early Detection

The first major shift came with the widespread adoption of consumer wearables capable of detecting rhythm irregularities. The Apple Heart Study enrolled nearly half a million participants and demonstrated that smartwatch-based photoplethysmography (PPG) could identify irregular pulse patterns with a low notification burden. Although the pragmatic design limited sensitivity estimates, the study established feasibility at scale. The Fitbit Heart Study [27] added younger adults and confirmed that remote, device-initiated detection workflows could be executed at a population level. Meta-analyses subsequently showed high diagnostic accuracy of smartwatch and single-lead ECG algorithms, validating their potential as first-line detection tools.

Soon after, randomized research emerged. For instance, the EQUAL study [37] in the Netherlands found that screening older adults using Apple Watches led to the detection of more atrial fibrillation—including cases that were paroxysmal or asymptomatic—compared to regular care. Similarly, the AMALFI trial [33], which sent ECG patches by mail, showed that remote, passive monitoring is also feasible. Taken together, these studies established wearable devices as scalable, non-invasive tools that can reveal a significant number of previously undiagnosed AF cases.

### 6.2. Integrating Digital Tools into Comprehensive AF Care

Digital health tools have expanded beyond detection into longitudinal care management. The mAFA-II trial exemplifies this shift: using a smartphone app that delivered the ABC pathway (anticoagulation, better symptom control, cardiovascular risk management), the intervention was associated with fewer adverse events in a multimorbid population [38,39]. The program illustrates how digital platforms can operationalize guideline-based care, support risk-factor modification, and coordinate follow-up within a unified framework. However, generalizability beyond selected patient groups and long-term effects on cognitive outcomes remain uncertain.

### 6.3. Medication Adherence and Real-World Challenges

Despite expanded detection, reduced stroke risk depends heavily on consistent anticoagulation. Real-world evidence indicates that nearly one-third of individuals on oral anticoagulants exhibit poor adherence, substantially elevating their risk of thromboembolic events. These observations motivated digital adherence strategies—automated reminders, extended prescription refills, personalized feedback, and behavioural nudges—particularly for older adults and other high-risk groups. Yet, data also reveal that even optimal adherence does not eliminate all residual risk, reinforcing the need for comprehensive, risk-factor-based management rather than narrow reliance on medication persistence alone.

### 6.4. Conversational AI and Virtual Support

Conversational AI has recently emerged as an adjunct to clinical care. The Spanish LOLATAO initiative [25], deployed within a haematology service, piloted a voice-based assistant that guided AF patients through medication management and routine followup. High user acceptability, reductions in missed doses, and enhanced clinician efficiency reflected the promise of such tools. Similar pilot and randomized studies in Australia and the UK tested virtual assistants in post-discharge and outpatient settings. While engagement and patient-reported usefulness were consistently high, larger trials are required to determine their effect on clinical outcomes.

### 6.5. Large-Scale Pragmatic Trials and Evidence Gaps

Major ongoing studies, including HEARTLINE, SAFER, and REACT-AF [41,42,43] aim to determine whether digital engagement accelerates diagnosis, improves anticoagulation persistence, and translates into measurable reductions in stroke, heart failure, or cognitive decline. Their findings will likely shape the next phase of digital AF care. Importantly, despite advances in detection and management, no trial has yet shown that digital interventions prevent cognitive decline or dementia in AF populations.

### 6.6. Equity, Usability, and Implementation Challenges

As digital platforms proliferate, equity has emerged as a critical theme. Older adults, individuals with cognitive impairment, and socially disadvantaged groups—those who may benefit the most—often encounter substantial barriers: limited digital literacy, reduced access to technology, and lower confidence using devices. Systematic reviews indicate that perceived control, social support, and tailored training are essential for sustained engagement. These considerations must be integrated into future design and implementation strategies to avoid widening existing health disparities [26,40,41].

### 6.7. Synthesis and Current Perspective

Across the AF care spectrum, digital health technologies have enhanced early detection, facilitated guideline-based management, and supported adherence. Their greatest promise—improving long-term cognitive outcomes—remains unproven. Observational studies suggest possible associations between improved AF management and reduced dementia risk, but the specific contribution of digital tools is still unclear. Additionally, concerns regarding usability, information overload, health equity, and unintended anxiety must be acknowledged.

In summary, digital health represents a powerful enabler of early AF diagnosis and integrated ABC-guided care. However, its role in protecting cognition and quality of life in vulnerable populations remains to be rigorously demonstrated. Future trials should prioritize older, multimorbid, and cognitively at-risk adults; incorporate detailed neurological endpoints; and explicitly address the digital divide to ensure equitable benefit [23,24,25,26,27,28,29,30,31,32,33,34,35,36,37,38,39,40,41,42,43]. The studies are summarized in Table 3.

## 7. Conclusions and Future Directions

Recent research suggests that digital health technologies are reshaping aspects of atrial fibrillation (AF) care, particularly in the areas of detection, monitoring, and the delivery of integrated management pathways. Large studies using smartwatch-based rhythm monitoring and ECG patches demonstrate that these tools can reliably identify AF in real-world settings and may help streamline patient evaluation and follow-up. When incorporated into structured care models such as the ABC pathway, mobile health solutions have been associated with improved adherence and reductions in selected cardiovascular outcomes. However, the strength of evidence linking digital health interventions to cognitive outcomes or quality-of-life improvements remains limited, with certainty generally rated as moderate to low. Current findings are influenced by reliance on observational data, short follow-up durations, underrepresentation of older and cognitively vulnerable populations, and persistent barriers such as digital literacy challenges, sensory or functional impairments, privacy concerns, and the need for caregiver support.

As the field evolves, future studies will need to evaluate digital health tools within rigorously designed, adequately powered randomized trials that include clearly defined cognitive endpoints and strategies adapted for older adults and those with cognitive difficulties. The conceptual understanding of AF has broadened, recognizing its potential contribution to brain injury through mechanisms beyond overt stroke—such as microembolism, hypoperfusion, microvascular disease, inflammation, and, in some circumstances, treatment-related intracranial bleeding. This expanded perspective has motivated calls for integrated, lifelong models of care that address both cardiovascular and neurological health. Digital technologies may help enable such models, but their capacity to confer brain-specific benefit remains to be demonstrated.

This shift toward a heart–brain framework reflects growing observational evidence associating AF with cognitive decline, even in the absence of clinically apparent stroke. Several therapeutic strategies—including anticoagulation, rhythm control, catheter ablation, and comprehensive risk-factor management—may be associated with more favourable cognitive trajectories, though the evidence is heterogeneous and largely observational. Anticoagulation, for example, is consistently linked to lower dementia risk, and some studies suggest a modest advantage of DOACs over VKAs. Nonetheless, these associations cannot establish causality, highlighting the urgent need for randomized trials specifically powered for cognitive outcomes. Likewise, while rhythm-control strategies and ablation have been associated with reduced dementia incidence in selected cohorts, confirmation in controlled trials—including a thorough assessment of procedural risks—remains essential.

Parallel progress in digital health has expanded opportunities for earlier AF detection and continuous remote monitoring. Smartwatch algorithms show high predictive value, and screening trials in older adults indicate that digital approaches can substantially increase AF detection. Conversational AI systems and virtual assistants have demonstrated high engagement and potential acceptability, particularly in anticoagulation management. Yet, direct evidence that these digital strategies reduce stroke, mortality, or cognitive decline is not yet available, and ongoing large-scale trials will be required to clarify their impact on clinically meaningful outcomes.

Looking forward, integrated digital platforms may offer a path toward more comprehensive models of AF management, linking continuous rhythm monitoring with cardiovascular, functional, and neurocognitive evaluations. Such systems could combine wearables or implantable monitors, automated anticoagulation support, home-based risk-factor tracking, cognitive assessments (including voice-based tools), targeted neuroimaging when appropriate, and coordinated care across cardiology and neurology. Early work suggests that these approaches are feasible, though their long-term clinical benefits, particularly regarding cognitive protection, require validation. Future research will need to incorporate inclusive design, caregiver support, and accessibility features to ensure equitable implementation across diverse patient populations.

Ultimately, managing AF as a condition with implications for both heart and brain health represents a promising direction for future care models. Combining pharmacological strategies, hemodynamic optimization, comprehensive risk-factor control, and digital support may help reduce the burden of AF-related complications. However, robust evidence is needed to confirm these potential benefits. Priorities for future investigation include randomized trials with cognitive endpoints, studies clarifying biological pathways linking AF to brain injury, development of biomarkers to identify individuals at greatest risk, and evaluation of integrated digital interventions in real-world settings. By addressing these evidence gaps, the field may advance toward strategies that more effectively protect both cardiovascular and cognitive health in individuals with AF.

## Figures and Tables

**Table 1:** Differences between AF phenotypes.

Measurement	Persistent AF	Paroxysmal AF	No AF
Total cerebral blood flow (mL/min)	472.1	512.3	541.0
Brain tissue perfusion (mL/100 g/min)	46.4	50.9	52.8

**Table 2:** Proinflammatory mediators elevated in both conditions.

Interleukin-1 (IL-1) Interleukin-8 (IL-8) Tumour necrosis factor-alpha (TNF-α) High-sensitivity C-reactive protein (hsCRP) Growth differentiation factor 15 (GDF-15)

**Table 3:** Summary of Major Trials and Digital Health Technologies in AF Management.

Study/Year	Design & Sample	Technology/Modality	Primary Endpoints	Cognitive/QoL Measures
Apple Heart Study (2019) [33]	Virtual, single arm, siteless pragmatic study N = 419,297 adults with Apple Watch (US)	Smartwatch photoplethysmography (PPG) for detection of irregular pulse Telehealth workflow + mailed ECG patch	Positive predictive value (PPV) for concurrent AF; proportion of notifications with AF confirmed on patch; notification burden	None (no cognitive or quality-of-life endpoints)
Fitbit Heart Study (2021/2022) [25]	Prospective, single-arm remote trial N = 455,699; median age 47 (US)	Smartwatch PPG for rhythm detection; 1-week ECG patch	PPV for concurrent AF (98.2%); AF yield on patch (~32%)	None (no cognitive or quality-of-life endpoints)
EQUAL (JACC 2025) [42]	Randomised controlled trial (RCT), Netherlands; 1:1 smartwatch versus usual care N = 437; ≥65 years; median CHA2DS2-VASc 3; 6-month follow-up	Apple Watch PPG + single-lead ECG integrated in telemonitoring workflow	New-onset AF detection increased (9.6% vs. 2.3%; hazard ratio 4.40); safety, emergency department visits, major adverse cardiovascular events (MACE) similar	None (no cognitive endpoints)
AMALFI (JAMA 2025) [31]	Remote, unblinded RCT in UK primary care; mail-out patch versus usual care N = 5040; ≥65 years; elevated CHA2DS2-VASc	14-day continuous ECG patch (mail-based)	Modest increase in AF diagnosis and anticoagulation exposure at ~2.5 years	None (no cognitive endpoints)
mAFA-II (JAMA Netw Open 2021; Win-ratio 2023) [36,37]	Cluster-randomised pragmatic programme (China) Core trial N ≈ 3324; multimorbidity analyses	mHealth app implementing ABC pathway (A: anticoagulation, B: symptom control, C: risk management)	Decrease in composite endpoint of stroke/thromboembolism, death, rehospitalisation; win-ratio 2.78	None (no cognitive endpoints reported)
HEARTLINE ([41])	Large pragmatic virtual study. N ≈ 34,244; ≥65 years with Medicare (US)	Apple Watch with irregular rhythm notification/ECG and app-based engagement; anticoagulation adherence module	Primary objectives: early AF diagnosis, cardiovascular outcomes, direct oral anticoagulant (DOAC) adherence; planned patient-reported outcomes	No cognitive endpoints publicly reported (to date)

## Data Availability

No new data were created or analysed in this study.

## Supplementary Materials

The following supporting information can be downloaded at: https://www.mdpi.com/article/10.3390/jcm15051744/s1. PRISMA checklist: PRISMA 2020 for Abstract Checklist.
